# Optimization of Using Multiple Machine Learning Approaches in Atrial Fibrillation Detection Based on a Large-Scale Data Set of 12-Lead Electrocardiograms: Cross-Sectional Study

**DOI:** 10.2196/47803

**Published:** 2024-03-11

**Authors:** Beau Bo-Sheng Chuang, Albert C Yang

**Affiliations:** 1 School of Medicine National Yang Ming Chiao Tung University Taipei Taiwan; 2 Digital Medicine and Smart Healthcare Research Center National Yang Ming Chiao Tung University Taipei Taiwan; 3 Department of Medical Research, Taipei Veterans General Hospital Taipei Taiwan

**Keywords:** machine learning, atrial fibrillation, light gradient boosting machine, power spectral density, digital health, electrocardiogram, machine learning algorithm, atrial fibrillation detection, real-time, detection, electrocardiography leads, clinical outcome

## Abstract

**Background:**

Atrial fibrillation (AF) represents a hazardous cardiac arrhythmia that significantly elevates the risk of stroke and heart failure. Despite its severity, its diagnosis largely relies on the proficiency of health care professionals. At present, the real-time identification of paroxysmal AF is hindered by the lack of automated techniques. Consequently, a highly effective machine learning algorithm specifically designed for AF detection could offer substantial clinical benefits. We hypothesized that machine learning algorithms have the potential to identify and extract features of AF with a high degree of accuracy, given the intricate and distinctive patterns present in electrocardiogram (ECG) recordings of AF.

**Objective:**

This study aims to develop a clinically valuable machine learning algorithm that can accurately detect AF and compare different leads’ performances of AF detection.

**Methods:**

We used 12-lead ECG recordings sourced from the 2020 PhysioNet Challenge data sets. The Welch method was used to extract power spectral features of the 12-lead ECGs within a frequency range of 0.083 to 24.92 Hz. Subsequently, various machine learning techniques were evaluated and optimized to classify sinus rhythm (SR) and AF based on these power spectral features. Furthermore, we compared the effects of different frequency subbands and different lead selections on machine learning performances.

**Results:**

The light gradient boosting machine (LightGBM) was found to be the most effective in classifying AF and SR, achieving an average *F*_1_-score of 0.988 across all ECG leads. Among the frequency subbands, the 0.083 to 4.92 Hz range yielded the highest *F*_1_-score of 0.985. In interlead comparisons, aVR had the highest performance (*F*_1_=0.993), with minimal differences observed between leads.

**Conclusions:**

In conclusion, this study successfully used machine learning methodologies, particularly the LightGBM model, to differentiate SR and AF based on power spectral features derived from 12-lead ECGs. The performance marked by an average *F*_1_-score of 0.988 and minimal interlead variation underscores the potential of machine learning algorithms to bolster real-time AF detection. This advancement could significantly improve patient care in intensive care units as well as facilitate remote monitoring through wearable devices, ultimately enhancing clinical outcomes.

## Introduction

Atrial fibrillation (AF) is the most prevalent cardiac arrhythmia, impacting an estimated 33.5 million people worldwide [1]. This severe cardiac condition heightens the risks of stroke and heart failure [2]. Clinicians typically detect and diagnose AF through the noninvasive electrocardiogram (ECG) method. However, ECG interpretation relies heavily on the expertise of the medical professional, creating a need for automated ECG classification to support clinicians. Machine learning, a subset of artificial intelligence, has shown great potential in improving the detection and management of AF through automated ECG analysis [3], risk stratification [4], or treatment planning [5].

The 2020 PhysioNet Challenge data sets offer 12-lead ECG recordings that can be used to evaluate machine learning techniques for ECG interpretation [6]. Compared to machine learning approaches based on small and homogeneous data sets, algorithms using PhysioNet data are likely to be more representative of realistic clinical scenarios, thereby making them better suited for practical implementation. Various machine learning techniques have been used to detect AF using ECG data. Some of the widely explored methods include support vector machines, decision trees, random forests, and deep learning approaches like convolutional neural networks and recurrent neural networks. These techniques have demonstrated promising results in classifying normal sinus rhythm (SR) and AF, with fair accuracy and *F*_1_-scores [4].

The performance of machine learning algorithms depends on the quality of the input features. Common feature extraction methods in AF detection include time-domain analysis, frequency-domain analysis, and wavelet transform. Power spectral density (PSD) is a popular frequency-domain feature that has been used to differentiate SR from AF. PSD analysis reveals information about the distribution of a signal’s power and frequency components. An ECG signal comprises various frequency components, including those related to the sinus heartbeat as well as atrial and ventricular activity. The PSD distribution of these frequency components may alter heart conditions that affect the cardiac contraction cycle, making it a potential indicator for identifying cardiac arrhythmias. Consequently, we developed an automated machine learning algorithm based on PSD to differentiate normal SR from AF based on a large-scale data set from PhysioNet 2020.

## Methods

### ECG Data

We used the 2020 PhysioNet Challenge data sets (Table 1) [6], comprising the China Physiological Signal Challenge (CPSC) Database (men: n=3699, women: n=3178, total: n=6877, and sampling rate: 500 Hz), CPSC-Extra Database (men: n=1843, women: n=1610, total: n=3453, and sampling rate: 500 Hz), St Petersburg Institute of Cardiological Technics INCART 12-lead Arrhythmia Database (total: n=72 and sampling rate: 257 Hz), Physikalisch Technische Bundesanstalt (PTB) Diagnostic ECG Database (men: n=377, women: n=139, total: n=516, and sampling rate: 1000 Hz), Physikalisch Technische Bundesanstalt extra large (PTB-XL) electrocardiography Database (men: n=11,379, women: n=10,458, total: n=21,837, and sampling rate: 500 Hz), and Georgia 12-lead ECG Challenge Database (men: n=5551, women: n=4793, total: n=10,344, and sampling rate: 500 Hz). The PhysioNet Challenge data sets can be accessed publicly [7], and data access is licensed under the Creative Commons Attribution 4.0 International Public License [8].

**Table 1:** 2020 PhysioNet challenge data sets.

Properties		Data set					
		CPSC^a^ Database	CPSC-Extra Database	St Petersburg INCART^b^ Database	PTB^c^ Diagnostic	PTB-XL^d^	Georgia
Recording time		6~60 seconds	6~60 seconds	30 minutes	Unknown	10 seconds	10 seconds
Sampling frequency (Hz)		500	500	257	1000	500	500
**Participants, n**							
	Male	3699	1843	N/A^e^	377	11,379	5551
	Female	3178	1610	N/A	139	10,458	4793
	Total	6877	3453	72	516	21,837	10,344

^a^CPSC: China Physiological Signal Challenge.

^b^INCART: Institute of Cardiological Technics.

^c^PTB: Physikalisch Technische Bundesanstalt.

^d^PTB-XL: Physikalisch Technische Bundesanstalt extra large.

^e^N/A: not applicable.

From these data sets, we initially selected data featuring a 500 Hz sampling rate. Next, we identified ECG diagnoses using the Systematized Nomenclature of Medicine—Clinical Terminology codes 426783006 and 164889003 for SR and AF, respectively. This led to the identification of 20,766 SR and 3458 AF ECG recordings. After excluding recordings corresponding to multiple diagnoses, the final data set comprised 9102 SR and 1088 AF ECG recordings.

### Ethical Considerations

This study was approved by the institutional review board of Taipei Veterans General Hospital (2022-04-004BC).

### ECG Preprocessing and Feature Extraction

**Figure 1:**
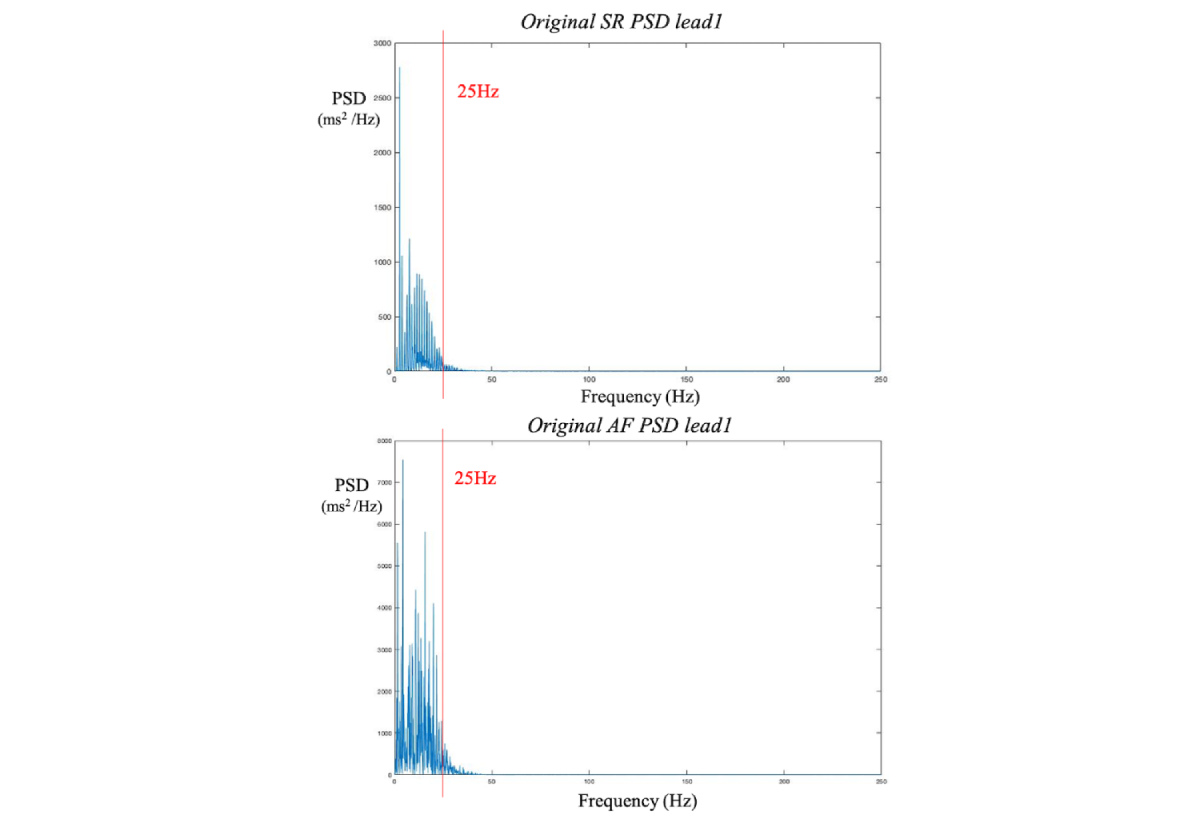
Initial results of PSD for SR and AF)in lead 1. The original frequency information we got from PSD is 0 to 250 Hz. From the figure, we can see that the power of the signal concentrates within 0 to 25 Hz. Thus, we selected 0 to 25 Hz as our main frequency band. AF: atrial fibrillation; PSD: power spectral density; SR: sinus rhythm.

**Figure 2:**
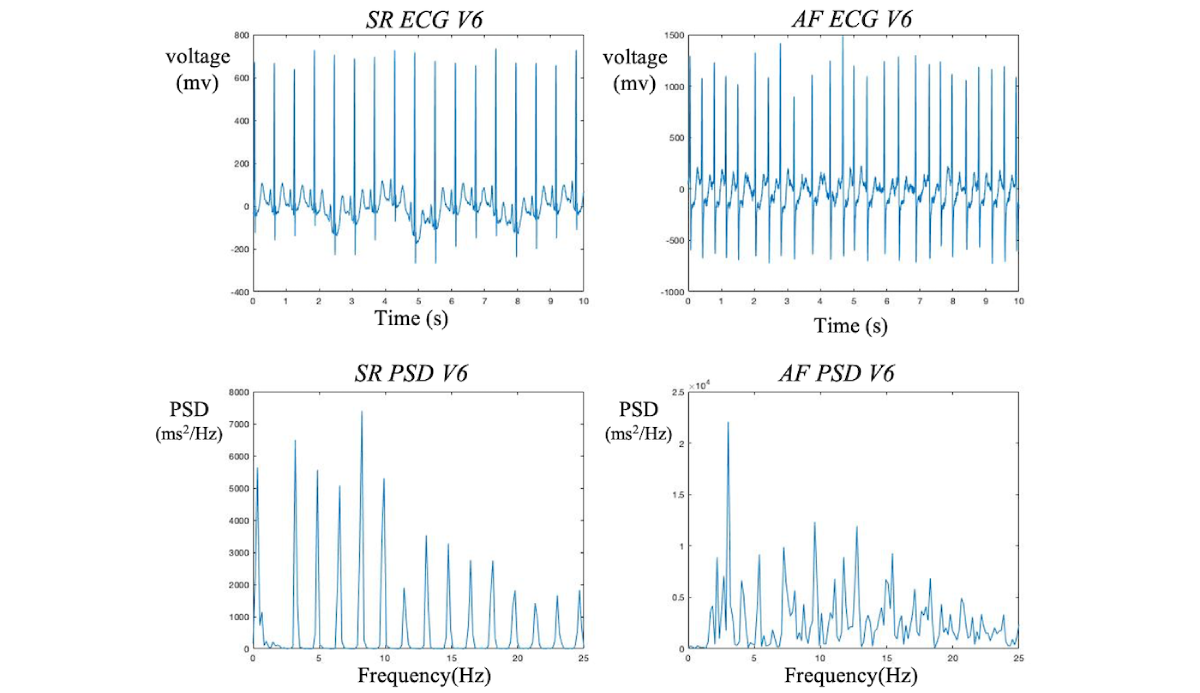
A comparison of the ECG and PSD for SR and AF in lead V4. While there is a discernible difference between SR and AF in the ECG representation, it remains challenging to distinguish them in this format. In contrast, the PSD figure demonstrates the harmonic nature of SR and the chaotic nature of AF, making it much easier to differentiate between the two. As a result of these observations, we have chosen to use PSD as the primary feature for our machine learning algorithms. This decision is based on the enhanced clarity and distinction provided by the PSD representation, which allows for more accurate and effective differentiation between SR and AF in our analysis. AF: atrial fibrillation; ECG: electrocardiogram; PSD: power spectral density; SR: sinus rhythm.

The ECG patterns associated with AF exhibit greater variability compared to those of SR. To explore this distinction, we used the Welch method [9,10] to estimate PSD and determine the frequency composition of the ECG signals. The recording durations of the ECG data ranged from 6 to 60 seconds, with 10 seconds being the most common. This variability could lead to differences in PSD resolution. To address this issue, we used the frequency bin method to ensure consistent frequency resolution across our PSD features. We set the frequency resolution to 1/6 Hz and calculated the arithmetic mean of the PSD frequencies within this resolution to represent the midpoint of each envelope. The data we used had a sampling rate of 500 Hz. Based on Nyquist-Shannon sampling theorem, the highest frequency information we can get is 250 Hz. After observing several data plots of the original PSD graph, we can see that most of the power of the signal is within 0 to 25 Hz. Thus, for a 10-second ECG recording, we selected a frequency of 0 to 25 Hz which was converted to 0.083 to 24.92 Hz. Figure 1 presents an example of original PSD plots and explains why we chose the PSD from 0 to 25 Hz. For further segmentation, it was just reasonable to separate the whole frequency segment into 5 smaller segments since the whole frequency ranges from 0 to 25 Hz. Figure 2 presents example ECG signals and PSD plots, highlighting notable differences in PSD between SR and AF; narrower harmonic frequency components can be observed in SR, while AF patterns display more erratic frequency components. We used these PSD features as inputs for our machine learning algorithm.

### Machine Learning Methods

Supervised machine learning is a type of machine learning where the algorithm is trained on a labeled data set, and the goal is to make predictions for new, unseen data. There are several types of supervised machine learning methods used in this study, including (1) discriminant analysis: a method for modeling the relationship between a dependent variable and one or more independent variables (used for continuous target variables); (2) logistic regression: a method for modeling the probability of a binary outcome based on one or more independent variables; (3) decision trees: a method for making predictions by creating a tree-like model of decisions and their potential consequences; (4) random forests: an ensemble method that combines multiple decision trees to make a prediction; (5) support vector machines: a method for classifying data by finding the best boundary (or “hyperplane”) that separates the classes; (6) neural networks: a method inspired by the structure and function of the human brain (can be used for a wide range of tasks, including classification and regression); and (7) naive Bayes: a probabilistic method for making predictions based on Bayes’ theorem (often used for text classification).

Additionally, we used a gradient boosting decision tree algorithm, namely light gradient boosting machine (LightGBM) [11]. It is one of the most efficient algorithms in recent years, known for its speed and accuracy. Boosting is an approach for combining multiple base models into a composite one. One of the ensemble tree algorithms of LightGBM is a “leaf-wise” tree algorithm, wherein the tree grows vertically; most of the others are “level-wise” tree algorithms, wherein the trees grow horizontally. The leaf-wise structure reduces time complexity and offers a favorable balance of accuracy and efficiency, especially for large-scale, high-dimensional data. Thus, it is useful in classification problems.

Perceptrons are single-neuron models and the precursors to larger neural networks; now, they are the building blocks of neural networks. A deep neural network (DNN) is a supplement of a feed-forward neural network and consists of 3 types of layers: the input layer, the output layer, and the hidden layer. The input layer receives the input signal to be processed. The output layer performs the required task, such as prediction or classification. The true processing engine is the hidden layers, placed between the input and output layers. In a DNN, data flow from the input layer to the output layer, just as in feed-forward neural networks. The neurons are trained through back propagation. DNNs are widely used in various applications, including automated diagnosis using ECG data [12].

### Statistical Analysis

#### Data Imbalance

We used the synthetic minority over-sampling technique [13] to filter the data and reduce the imbalance between the numbers of SR and AF recordings (n=9102 and n=1088, respectively).

#### *F*_1_-Score

To mitigate bias resulting from data imbalance, we used the *F*_1_-score as the primary metric for evaluating machine learning models. *F*_1_-scores treat false-positive and false-negative errors equally and are more useful than accuracy in cases of class imbalance. The *F*_1_-score is the harmonic mean of precision and recall, which measures the errors contributed by false positives and false negatives, respectively.

#### Training, Validation, and Testing Data Sets

We divided our data sets into training, validation, and testing sets. The training set was used to develop the models, the validation set for model tuning, and the testing set for model assessment. In this study, 60% of the data comprised the training set, 20% the validation set, and 20% the testing set. For model tuning, we used ensemble learning to identify optimal parameters for each model based on the validation set.

## Results

### Comparison of the Performance of Various Machine Learning Algorithms

We evaluated various machine learning methods, including extra tree, LightGBM (Microsoft Corporation), CatBoost (Yandex), XGBoost (The XGBoost Contributors), decision tree, k-nearest neighbors, stochastic gradient descent, gradient boosting, random forest, naïve Bayesian, logistic regression, and DNN. Our analysis reveals that machine learning algorithms with boosting methods outperform those without boosting. In particular, LightGBM outperformed the other methods, achieving the highest average *F*_1_-score of 0.988 across all 12 ECG leads and the lowest computation time for features within the entire frequency range (0.083-24.92 Hz). Conversely, our results indicate that the naive Bayesian algorithm performs poorly in classifying SR and AF. Despite its simplicity, naive Bayesian appears to be less suited for this specific task, and alternative approaches should be considered for better accuracy and reliability. Thus, we focused solely on LightGBM for subsequent analyses.

### Effect of Frequency Band on Model Performance

We examined the contributions of various frequency subbands of ECG PSD features to model performance in detecting AF (Table 2). The entire frequency range was divided into 5 subbands (0.083-4.92, 5.083-9.92, 10.083-14.92, 15.083-19.92, and 20.083-24.92 Hz). The highest overall *F*_1_-score was achieved by the model using the full frequency range (0.083-24.92 Hz), and the frequency subband of 0.083 to 4.92 Hz yielded the highest *F*_1_-score among the subbands. Generally, the *F*_1_-score for each subband was around 0.9, indicating that every subband contains valuable information for ECG classification.

**Table 2:** *F*_1_-scores for various frequency bands.

Performance	Frequency range					
	Whole range	0.083-4.92 Hz	5.083-9.92 Hz	10.083-14.92 Hz	15.083-19.92 Hz	20.083-24.92 Hz
*F*_1_-score	0.988	0.985	0.956	0.925	0.912	0.900

### Interlead Differences in Model Performance

In lead comparisons, aVR demonstrated the highest performance across all frequency subbands, although the differences between leads were minimal (Table 3). Specifically, for the entire frequency range, using aVL resulted in the lowest classification performance (*F*_1_-score=0.980), while using aVR achieved the highest performance (*F*_1_-score=0.993).

**Table 3:** *F*_1_-scores for various leads and frequency bands.

Frequency range	Different leads											
	l1	l2	l3	aVR	aVL	aVF	V1	V2	V3	V4	V5	V6
Whole range	0.985	0.990	0.981	0.993	0.980	0.984	0.988	0.988	0.991	0.991	0.990	0.989
0.083-4.92 Hz	0.980	0.989	0.978	0.990	0.980	0.980	0.986	0.988	0.987	0.990	0.990	0.987
5.083-9.92 Hz	0.961	0.968	0.930	0.978	0.936	0.957	0.961	0.947	0.959	0.958	0.963	0.958
10.083-14.92 Hz	0.920	0.928	0.900	0.949	0.917	0.921	0.926	0.923	0.936	0.940	0.912	0.937
15.083-19.92 Hz	0.878	0.915	0.854	0.948	0.900	0.909	0.920	0.907	0.923	0.927	0.938	0.924
20.083-24.92 Hz	0.868	0.915	0.857	0.939	0.871	0.885	0.877	0.882	0.920	0.916	0.940	0.925

## Discussion

### Principal Findings

In this study, we found that the LightGBM algorithm was the most effective machine learning model for our purposes. Notably, all frequency subbands contained distinct information, achieving independent *F*_1_-scores of approximately 0.9. Consequently, using the entire frequency range (0.083-24.92 Hz) provided the most comprehensive features and yielded the highest *F*_1_-score of 0.988. Interestingly, among the limb leads, aVR produced the best result, which was marginally superior to the outcomes of chest leads and lead II. This finding is unexpected, as lead aVR is rarely used in real-world scenarios for detecting arrhythmia. The contributions of different frequency bands to classification performance showed that even though each subband contains unique features, the dominant frequency subband (0.083-4.92 Hz) is sufficient for distinguishing AF from SR.

In terms of the contributions of frequency bands to classification performance, it is important to note that the normal human heart beats between 60 to 100 times per minute (1-1.67 Hz), whereas the dominant frequency in AF ranges from 3.8 to 8 Hz [14]. Given the differences in frequency components between SR and AF, they can be used to distinguish one from the other. As demonstrated in Figure 1, multiple frequency peaks are observed within the 0 to 25 Hz range for both SR and AF. Consequently, frequency subbands containing distinct features may yield different results.

Initially, we hypothesized that the subband containing the dominant frequency (0.083-4.92 Hz) would result in the optimal classification of SR and AF, similar to the whole frequency range (0.083-24.92 Hz), while other subbands would struggle to classify SR and AF effectively. Indeed, models using the dominant frequency subband performed well, with results closely resembling those of models using the whole frequency range. However, other subbands also demonstrated high performance independently (Table 2), with *F*_1_-scores exceeding 0.854. The dominant frequency subband (0.083-4.92 Hz) yielded an *F*_1_-score of 0.985, which is comparable to that of the whole frequency range (*F*_1_-score=0.988). These findings suggest that although each subband contains unique features and cannot be replaced by other subbands, the dominant frequency subband alone is sufficient for differentiating AF from SR.

In this study, we explore the variations in AF classification performance across different ECG leads. Each ECG lead has unique physiological applications, which in turn influence the preference of medical professionals in identifying specific types of arrhythmias. For instance, lead 1, aVF, or V1 are frequently used in diagnosing right ventricular hypertrophy. Routine clinical practice suggests that lead 2, which is typically the focus of medical professionals when examining AF, would yield the most accurate results. Surprisingly, our findings revealed that all leads, within the frequency range of 0.083-24.92 Hz, generated *F*_1_-scores in close proximity to 0.985. Among these, lead aVR achieved the highest *F*_1_-score at 0.993. We believe this can be attributed to the visibility of AF in the majority of leads and the comprehensive features encompassed by the entire frequency range.

Contrary to our initial expectations, lead aVR, which is not a common choice in clinical settings, produced the most optimal results when using subbands [15]. This finding may potentially shed light on the reentry mechanism that underlies AF [16,17]. It is worth noting that lead aVR captures data from the right ventricle outflow tract and the basal portion of the septum, whereas AF predominantly manifests in the left ventricle. Consequently, the reentry mechanism emerges as the most plausible explanation for this observed phenomenon.

In a prior study [18], the 2020 PhysioNet Challenge data sets were used alongside the LightGBM machine learning algorithm. However, the approaches to signal extraction and processing in that study differed from those used in our research. The previous study implemented filtering techniques and wavelet multiresolution analysis, while our investigation used PSD and the Welch method. Another key distinction between the 2 studies is the scope of the classification task. While our research focused solely on differentiating AF from SR, the previous study attempted to identify 24 different diseases. Due to the varied physiological information provided by each ECG lead, we opted to compare the performance of LightGBM for each individual lead, rather than inputting data from all 12 leads into the LightGBM simultaneously. Furthermore, our study identified the most critical frequency subband within the total frequency range. We determined the dominant frequency subband to be 0.083-4.92 Hz, which played a significant role in our analysis.

There are several limitations to be addressed in this study. One notable constraint is our selection of 10-second ECG recordings, which may not accurately represent the full spectrum of real-world clinical scenarios. The data sets used in this study were predominantly clean, while actual clinical situations may involve data contaminated by noise, potentially affecting the results. Moreover, our study focused exclusively on single-diagnosis data, representing either AF or SR. This approach, however, does not account for the possibility of a patient experiencing multiple arrhythmias concurrently. Consequently, our findings were derived under simplified conditions that may not fully reflect the complexity of real-life cases. In light of these limitations, it is essential to interpret our results with caution and consider further research that incorporates a broader range of ECG recordings, addresses potential noise-related challenges, and examines cases with multiple coexisting arrhythmias to enhance the generalizability and applicability of our findings to real-world clinical settings.

In summary, LightGBM proves to be a highly effective algorithm for distinguishing AF from SR. Our study demonstrated that when dividing the entire ECG frequency range into subbands with fewer features, lead aVR delivered the best performance. This outcome could potentially be associated with the underlying pathological mechanisms of AF. By incorporating adequate frequency band features, lead 2 ECG data can achieve an *F*_1_-score of approximately 0.99. This level of accuracy and efficiency of the LightGBM model renders the algorithm suitable for implementation in clinical practice and integration into commercialized electronic devices, such as the smartwatch with the ECG functionality, for a range of health care applications. This study’s findings suggest that leveraging the power of LightGBM could enhance arrhythmia detection and monitoring, ultimately improving patient care and outcomes.

### Literature Review

In this review, we will talk about studies that introduce techniques for AF detection, analyzing their methods, results, and potential implications in the field of cardiology. First, one study [19] proposed a feature extraction method based on a gradient set for AF detection, which features simplicity, noise tolerance, and adaptability to various classifiers. However, we can still improve the feature extraction method in the future, such as improving AF detection performance by proposing a more representative feature set or combining it with other types of features. This approach shows the potential for streamlined, efficient feature extraction, providing a solid foundation for further research in the domain.

Next, the Transposed Projection–Convolutional Neural Network (TP-CNN) method was introduced in another study [20], aiming to use “compressed ECG signals” for AF detection. The approach demonstrates promising results in accurately detecting AF in wearable application scenarios while addressing energy consumption concerns. It shows the effective use of compressed signals and a highly accurate algorithm. Yet, its data sources only contain 25 patients. More patients should be included in further studies.

Another study [21] demonstrates a unique method using multiple parameters, including the average number of f waves in a TQ interval, showing robust real-time AF detection capabilities. This approach not only differentiates between AF and normal ECGs but also outperforms distinguishing AF from other arrhythmias. However, they can discuss more about how their algorithms will be implemented in clinical situations.

There is another study [22] focusing on low-complexity algorithms for AF detection, a method based on RR interval features. This approach demonstrated reasonable feature selection while maintaining a low computational cost, making it suitable for low-power devices. However, in future studies, it should focus on getting a better *F*_1_-score.

In addition to ECG AF detection, there is also a signal called photoplethysmography common in wearable devices for AF detection, while we usually use ECG in clinical situations. This Fitbit Heart study [23] provides an algorithm that exhibited a high positive predictive value for concurrent AF, highlighting the potential of consumer wearables for large-scale AF identification. Though the result is nice, we hope we can know more about photoplethysmography comparison with ECG.

In conclusion, these studies collectively contribute to the evolving landscape of AF detection methods. While each approach offers unique advantages, further research should focus on refining these methods, exploring real-time applications, and enhancing the overall accuracy and efficiency of AF detection algorithms. Potential limitations should also be emphasized in future studies.

## Abbreviations

AF: atrial fibrillation
CPSC: China Physiological Signal Challenge
DNN: deep neural network
ECG: electrocardiogram
INCART: Institute of Cardiological Technics
LightGBM: light gradient boosting machine
PSD: power spectral density
PTB: Physikalisch Technische Bundesanstalt
PTB-XL: Physikalisch Technische Bundesanstalt extra large
SR: sinus rhythm
TP-CNN: Transposed Projection–Convolutional Neural Network

## Data Availability

The data sets generated and analyzed during the current study are available in the 2020 PhysioNet Challenge data sets repository [7].
